# A young man with myelosuppression caused by clindamycin: a case report

**DOI:** 10.1186/1752-1947-8-7

**Published:** 2014-01-05

**Authors:** Manuel Polanco Morales, Anna Paola Thome Carvallo, Karla Adriana Bautista Espinosa, Edgar Enrique Meza Murillo

**Affiliations:** 1 Department of Hematology and Bone Marrow Transplantation, The American British Cowdray Medical Center, Sur 136, número 116, colonia Las Américas, Delegación Álvaro Obregón, C.P. 01120, México, D.F., Mexico; 2 Department of Internal Medicine, The American British Cowdray Medical Center, Sur 136, número 116, colonia Las Américas, Delegación Álvaro Obregón, C.P. 01120, México, D.F., Mexico

**Keywords:** Clindamycin, Myelosuppression, Pancytopenia, Side effects

## Abstract

**Introduction:**

Clindamycin is used to treat various bacterial infections, but its administration can cause anaphylaxis, liver reactions, pseudomembranous colitis, and peripheral blood cytopenias (anemia, neutropenia, and thrombocytopenia), alone or in combination. We report the case of a patient with a recurrent infection of the tonsils who received clindamycin. Pancytopenia, a previously unreported hematological disorder related to clindamycin use, was observed in conjunction with the infection and clindamycin treatment.

**Case presentation:**

One month prior to hospitalization, a 22-year-old man of Hispanic origin had a tonsillar infection and cough and began to have anal pain. These conditions became exacerbated three weeks later, coinciding with a new tonsillar infection, frequent nonproductive cough, and febrile syndrome. He received clindamycin for four days prior to his admission, without improvement. While hospitalized, he was found to have fever, tonsillar abscess, hemorrhoid thrombosis, and anal fissure; the latter was immediately resected under general anesthesia. Before surgery, our patient’s blood count showed intense leukoneutropenia and mild thrombocytopenia that increased 12 hours later, along with the establishment of anemia. A bone marrow study showed decreased cell content, micromegakaryocytes, and an interruption of the differentiation of granulocytes and erythroblasts. Post-surgery, our patient received metronidazole, meropenem, and amikacin along with acetaminophen, ketoprofen, omeprazole, and pegfilgrastim, with resulting clinical and hematological improvement.

**Conclusion:**

Our experience with this patient establishes that well-documented clinical cases should be the basis for identifying and publicizing unknown or uncommon undesirable effects of drugs. We report that, in some individuals, clindamycin can cause pancytopenia, a complication that in our patient’s case was caused by direct injury of his hematopoietic tissue.

## Introduction

Clindamycin alters ribosomal function and reversibly inhibits bacterial protein synthesis. It is successfully and safely used to treat infections by various microorganisms [1]. However, several undesirable effects have also been described, such as isolated or combined cytopenias in the peripheral blood [1-4].

**Figure 1:**
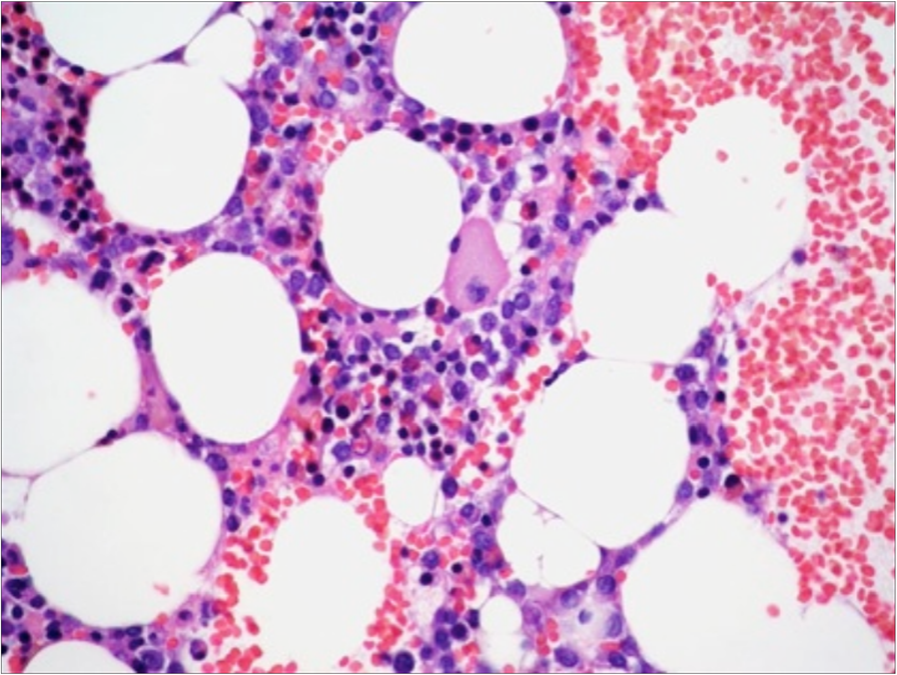
**Bone marrow biopsy of iliac crest.** Image shows diminished cell content; scarce megakaryocytes, with the sole one observed hypolobulated; and diminution of mature granulocytes and erythroid precursors. Note the relative increase of lymphocytes and plasma cells. Hematoxylin and eosin stain; original magnification × 40.

We report the clinical case of a patient who received clindamycin for recurrent infection of his tonsils. Because our patient required intensive treatment for the infection and surgical treatment for simultaneous proctological manifestations, he was hospitalized. Intense leukoneutropenia and mild thrombocytopenia were found preoperatively (Figure 1) and intensified in the immediate postoperative period, when anemia was also found.

We present the previously unreported presence of pancytopenia in connection with clindamycin use and we discuss its physiopathogenesis, progression, and treatment. We provide a review of the literature related to blood disorders attributable to clindamycin use.

## Case presentation

Our patient was a 22-year-old Hispanic man with no significant personal medical history. One month before treatment, he developed an irritating cough and tonsillitis, which were treated with 500mg of amoxicillin per day for seven days and 500mg of acetaminophen every six hours for nine days. He improved but continued to have a cough, which was controlled with irregular use of ambroxol and clenbuterol. Along with the aforementioned infection, our patient began to experience anal pain. Three weeks later, this increased in intensity, coinciding with a new tonsillar infection, a very frequent nonproductive cough and a fever of 39°C. To treat this, four days prior to being admitted to our hospital, our patient took 1,800mg/day of clindamycin.

After admission and upon examination, our patient had a fever of 38.6°C, a heart rate of 97 beats/min, a respiratory rate of 20 breaths/min, and blood pressure of 124/68mmHg. He weighed 69kg and was 1.67m in height. His general condition was good: he was conscious and cooperative, with normal mobility. He had pharyngeal erythema and hypertrophy of his tonsils with purulent discharge on their surface. We also found a painless 2 × 1cm ganglion under the angle of his left maxillary. A thoracoabdominal examination was normal; purpura and bone pain were not found.

Upon admission, laboratory tests revealed a normal red blood cell count, intense leukoneutropenia, and incipient thrombocytopenia (Figure 1). His erythrocyte sedimentation rate was 45mm/hour (normal: 0 to 20mm/hour), and his C-reactive protein level was 12.2mg/dL (normal: 0 to 0.3mg/dL). Levels of glucose (78mg/dL), blood urea nitrogen (10mg/dL), and creatinine (1.0mg/dL) were normal; liver function tests and coagulation studies were also normal. Immediately after admission, we performed an anal examination under general anesthesia. We found a hemorrhoid thrombosis and an anal fissure, which were resected without incident.

On the first day of hospitalization, our patient’s intense leukoneutropenia continued, his thrombocytopenia worsened (Figure 1), and his hematocrit (Ht) decreased to 36.2%. Determinations of vitamin B_12_, folic acid, and beta-2-microglobulin were normal. Immunofluorescence showed he was negative for antinuclear antibodies, as well as anti-double-stranded deoxyribonucleic acid (DNA), venereal disease research laboratory test, heterophile antibodies, anti-human immunodeficiency virus (HIV) 1 and 2, anti-toxoplasma, rubella, cytomegalovirus, herpes simplex virus 1 and 2, and parvovirus B19. Serologic tests for hepatitis were negative, except for the antibody against the S antigen of the B virus, but our patient had received the hepatitis B vaccine a year earlier. Neck and thoracoabdominal tomography showed no adenopathies, visceromegalies, or space-occupying lesions, while a tonsil culture and two blood cultures were negative.

**Figure 2:**
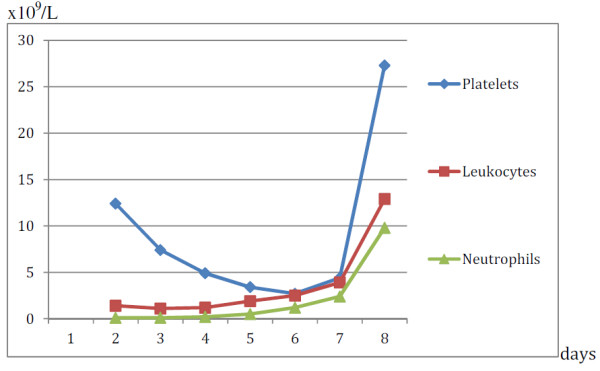
**Evolution of platelets (×10**^**10**^**/L), leukocytes, and neutrophil (×10**^**9**^**/L) values.**

Because of his progressive pancytopenia, we conducted aspiration and biopsy of the bone marrow in his iliac bone on the first day of hospitalization. In the aspirate, the cell content was extremely decreased at barely 25% of normal. Our patient had scarce hematopoietic tissue; we observed scarce megakaryocytes, some small and unlobed. A differential cell count showed an increased lymphocyte percentage (48.80%); the myeloid:erythroid relationship, with both cell lines diminished, was 2:1. The myeloid component (27% of the total) was formed by promyelocytes (8%) and myelocytes (8%), indicating the stage at which differentiation was interrupted. The metamyelocytes accounted for 4%, banded neutrophils for 3%, and segmented neutrophils for 2%. We found megaloblastic changes in this cell line, as well as pyknosis in the early forms of differentiation, increased cytoplasmic granulation, and, in some elements, vacuoles. The erythroid series was decreased to 12% and showed pyknosis and disruption of differentiation at the intermediate stages. In the bone biopsy, the decreased cellular content (Figure 2) was characterized by 3 megakaryocytes/mm^2^, increased lymphocytes and polyclonal plasma cells, and a normal number (5%) of CD 34+ cells, with grade 1 reticulin fibrosis.

Regarding the evolution of our patient’s symptoms, during the first day of hospitalization his fever was continuous and reached 39°C; on the second day, it became intermittent; and it disappeared on the third day. However, he continued to present with odynophagia and cough with whitish expectoration that gradually subsided. Post-surgery, he had diminishing anal pain and intermittent bloody drainage, which disappeared after two days.

Starting during the preoperative period, our patient received 500mg of intravenous metronidazole every eight hours for eight days. After surgery, meropenem (1g every eight hours) and amikacin (500mg every 12 hours) were added to his therapy for six days. As prophylactics, he was given fluconazole (100mg every 12 hours) and oral nystatin for five days. Paracetamol was also administered as an antipyretic, and ketoprofen was given as an analgesic. To control coughing, our patient received benzonatate, ipratropium bromide, and budesonide, and to prevent gastritis, he received omeprazole. As a result of the results from the bone marrow study, 6mg of pegfilgrastim (peg-G-CSF [recombinant human granulocyte colony-stimulating factor]) were given.

On the fourth day of hospitalization, our patient continued to present odynophagia, oropharyngeal hyperemia, and amygdalin secretion, but these disappeared during the course of the day, as did the axillary adenopathy. That night, he had a transient fever of 38°C caused by phlebitis secondary to the intravenous infusion. The next day, he reported pain in the long bones of his limbs, which was attributed to peg-G-CSF.

On that day, our patient’s blood counts showed increased Ht to 44%, increased absolute leukocytes and granulocytes, and further diminished platelet counts (Figure 1), without bleeding or purpura. On his seventh hospitalization day his platelet count began to increase. Our patient was discharged on the 13th day of hospitalization with mild leukocytosis and neutrophilia, and normal platelets and Ht. After three months of follow-up, we found no abnormalities in his blood cell counts.

Our patient presented with recurrent tonsillitis and developed pancytopenia in his peripheral blood because of the histopathological lesion of bone marrow which was chronologically related to clindamycin exposure. This relationship can be established by virtue of the time that elapsed between the use of the medication, the documentation of pancytopenia [5], and the finding of the alterations described in his bone marrow.

It has already been mentioned that undesirable hematological effects, such as anemia, neutropenia, and thrombocytopenia, can occur with the use of clindamycin, alone or in combination [1-4]. The effects are not frequent, and to the best of our knowledge, pancytopenia had not previously been reported. In the case of the first-mentioned cytopenias, their physiopathogeneses have been attributed to hemolysis in cases of anemia [2]. Cases of neutropenia may be caused by direct damage to the neutrophils or their precursors, inhibition of granulocyte colony-stimulating factor (G-CSF) activity, or an idiosyncratic reaction [6,7]. Thrombocytopenia may be caused by the immune destruction of platelets; as in cases of neutropenia, this can be caused by an idiosyncratic reaction [8-10]. It is interesting to note that none of the reports of these manifestations has included a study of the bone marrow tissue. Thus, our patient is particularly interesting because the pancytopenia was established by injury of the hematopoietic tissue (Figure 2).

Regarding the neutropenia, Bubalo *et al*. [3] reported the case of a patient with this condition in 2003. As of that time, only three other cases had been reported in the literature; however, those studies questioned whether clindamycin was the causative agent. In 2012, Schmidt and Reiter [4] reported the case of a patient with neutropenia who, undergoing major head and neck reconstructive surgery, received 900mg of intravenous clindamycin every six hours for one day preoperatively and every eight hours postoperatively for prophylactic purposes. After four days of clindamycin use, their patient’s leukocytes abruptly decreased to 1.1 × 10^9^ cells/L, and their absolute neutrophil count decreased to 165 cells/μl. Considering that there was no other possible cause responsible for this anomaly, clindamycin was suspended, and filgrastim was administered daily until the neutrophil count returned to normal four days later. The authors attributed the abnormality to G-CSF inhibition or direct toxicity of the neutrophils, without showing any evidence of either cause.

Furthermore, it is accepted that many drugs can cause thrombocytopenia via the immune destruction of platelets or the suppression of thrombopoiesis [8-10]. Immune destruction can occur two to five days after exposure to different drugs, and although clindamycin appears on the long list of drugs capable of causing thrombocytopenia via immune destruction or other mechanisms [8-11], we find no reference to support that process.

Something similar happens with anemia because we were only able to find an abstract in English of a paper published by Xuxia and Hongling [2] in the Chinese medical literature. A 37-year-old patient experienced severe anemia from hemolysis after 24 hours of receiving 3g per day of intravenous clindamycin. The abstract does not mention the patient’s physiopathogenesis, but Wilkin and Feinberg in 1999 [12] reported that clindamycin causes anemia 'sometimes by hemolysis,’ without alluding to other mechanisms. It is interesting to note that we did not find new references to this complication in the medical literature.

Finally, Safrin *et al.*[13] studied 58 patients with acquired immune deficiency syndrome (AIDS) and *Pneumocystis carinii* pneumonia treated with a combination of clindamycin and primaquine. They observed neutropenia in 14% of patients, anemia in 10%, and thrombocytopenia in 7%; complications were grade III-IV in 12% of patients, which prevented further treatment. With these data, the authors proposed not using clindamycin in patients with previous blood count abnormalities. However, it should be noted that, in patients with acquired immune deficiency syndrome (AIDS), cytopenias may be a manifestation of the disease or its complications. Also, these patients experience hypersensitivity reactions to drugs, particularly clindamycin, more often than the general population, and primaquine may cause hemolysis in susceptible individuals. These factors may explain the frequency and intensity of cytopenias in this particular group of patients [14].

In accordance with what has been mentioned, it can be established that the incidence of unwanted hematological effects attributable to clindamycin is very low and that, in general terms, the mechanism behind these effects is unknown. This case report is of special interest, because it illustrates that, in our patient, pancytopenia resulted from direct injury of the hematopoietic cells [5,15]. This was evident from their decreased numbers, morphological alterations, and interference with their differentiation in bone marrow (Figure 2). In addition, the pancytopenia recovered when clindamycin use was interrupted and peg-G-CSF was administered (Figure 1), and the amygdalin infection was adequately controlled.

Regarding the treatment of pancytopenia, because our patient developed a surgical wound in a naturally contaminated zone and a difficult-to-control tonsillar infection, the presence of the lesion documented in bone marrow (Figures 1 and 2) led to the use of peg-G-CSF in addition to antibiotics and symptom management drugs, which undoubtedly accelerated the recovery.

## Conclusion

We report the case of an individual who received clindamycin to treat a recurrent tonsillar infection. Coincident with the infection and the use of clindamycin, he developed pancytopenia in his peripheral blood (Figure 1), caused by direct damage of the hematopoietic tissue (Figure 2). The intimate mechanism of this injury was not investigated, but it may be the result of an idiosyncratic reaction, understood as a mixture of allergic and immunological phenomena caused by the clindamycin and perhaps facilitated by the infectious process that led to its use [5,15].

According to previous publications, it can be established that undesired hematologic reactions to clindamycin are exceptional, but they can be intense and have serious repercussions, especially if the physiopathogenesis passes undetected.

It is important to consider that, in the absence of clinical and/or epidemiological information, clinical case reports of adverse drug reactions such as this one form the basis for identifying and disseminating the rare risks of drugs.

## Consent

Written informed consent was obtained from the patient for publication of this case report and any accompanying images. A copy of the written consent is available for review by the Editor-in-Chief of this journal.

## Abbreviations

G-CSF: Granulocyte colony-stimulating factor; Ht: Hematocrit; Peg-G-CSF: Pegfilgrastim.

## Competing interests

The authors declare that they have no competing interests.

## Authors' contributions

MPM and MME were the hematologists in charge of the patient. MME and EBKA where the major contributors in writing the manuscript. CTP was the primary attending physician. All authors read and approved the final manuscript.
